# Random coil chemical shifts for serine, threonine and tyrosine phosphorylation over a broad pH range

**DOI:** 10.1007/s10858-019-00283-z

**Published:** 2019-10-09

**Authors:** Ruth Hendus-Altenburger, Catarina B. Fernandes, Katrine Bugge, Micha B. A. Kunze, Wouter Boomsma, Birthe B. Kragelund

**Affiliations:** 1 Structural Biology and NMR Laboratory, Department of Biology, University of Copenhagen, Ole Maaløes Vej 5, 2200 Copenhagen N, Denmark; 2 Department of Computer Science, University of Copenhagen, Universitetsparken 1, 2100 Copenhagen Ø, Denmark

**Keywords:** Secondary structure, NMR, Phosphorylation, Post translational modification, Secondary chemical shift analysis, PTM, IDP, Random coil

## Abstract

Phosphorylation is one of the main regulators of cellular signaling typically occurring in flexible parts of folded proteins and in intrinsically disordered regions. It can have distinct effects on the chemical environment as well as on the structural properties near the modification site. Secondary chemical shift analysis is the main NMR method for detection of transiently formed secondary structure in intrinsically disordered proteins (IDPs) and the reliability of the analysis depends on an appropriate choice of random coil model. Random coil chemical shifts and sequence correction factors were previously determined for an Ac-QQXQQ-NH_2_-peptide series with X being any of the 20 common amino acids. However, a matching dataset on the phosphorylated states has so far only been incompletely determined or determined only at a single pH value. Here we extend the database by the addition of the random coil chemical shifts of the phosphorylated states of serine, threonine and tyrosine measured over a range of pH values covering the pKas of the phosphates and at several temperatures (www.bio.ku.dk/sbinlab/randomcoil). The combined results allow for accurate random coil chemical shift determination of phosphorylated regions at any pH and temperature, minimizing systematic biases of the secondary chemical shifts. Comparison of chemical shifts using random coil sets with and without inclusion of the phosphoryl group, revealed under/over estimations of helicity of up to 33%. The expanded set of random coil values will improve the reliability in detection and quantification of transient secondary structure in phosphorylation-modified IDPs.

**Electronic supplementary material:**

The online version of this article (10.1007/s10858-019-00283-z) contains supplementary material, which is available to authorized users.

## Introduction

Intrinsically disordered proteins (IDPs) are important components of the cellular signaling machinery (Wright and Dyson 2015) and they are abundant in most proteomes (Ward et al. 2004; Xue et al. 2012). They exist as an ensemble of interconverting dynamic conformations with varying amounts of transiently populated secondary structure. Phosphorylation mostly occurs in intrinsically disordered regions (Iakoucheva et al. 2004; Tyanova et al. 2013), and can have diverse effects on transiently populated secondary structures. In the literature, cases can be found where there is no effect on the secondary structure (Sibille et al. 2012), but also cases where varying degrees of (de)stabilization are seen (Miranda et al. 2004; Espinoza-Fonseca et al. 2008; Andrew et al. 2002; Bui and Gsponer 2014), depending in most instances on the immediate sequence context (Hendus-Altenburger et al. 2017). One of the most pronounced effects reported is the phosphorylation-induced global folding of the IDP 4EBP2, which is the major neural isoform of a family of three mammalian proteins that bind eIF4E and suppress cap-dependent translation initiation (Bah et al. 2015).

The most convenient and robust NMR method to identify secondary structures in proteins is based on the secondary chemical shift analysis (SCS, Δδ). Chemical shifts (δs, CSs) of the backbone nuclei and in particular those of H^α^, C^α^ and C′ correlate strongly with local backbone structure. By comparison to random coil CSs, one can derive secondary structure propensities and specifically identify the position, length and population of these in proteins. Yet, random coil CSs of individual residues vary depending on their neighboring residues (Wishart et al. 1995) as well as on experimental conditions (salt, pH, temperature etc.) (Kjaergaard et al. 2011; Nielsen and Mulder 2018). This is critical, especially for IDPs as the transient nature of their secondary structure manifests in small SCSs, which means that small variations in the reference random coil libraries can lead to large biases. Currently, several datasets for reference CSs of commonly occurring amino acids exist that are based on either neighboring correction factors derived from peptide libraries (Kjaergaard et al. 2011; Kjaergaard and Poulsen 2011; Marsh et al. 2006), computational approaches (Camilloni et al. 2012), or curated CS data sets derived from disordered proteins (Nielsen and Mulder 2018; Tamiola et al. 2010). Posttranslational modifications (PTMs) affect the CSs, and the chemical effects of PTMs, like phosphorylation, on the random coil CSs are not yet included in any library. Previous studies have aimed to characterize the random coil CSs of phosphorylated amino acids using peptides (Bienkiewicz and Lumb 1999; Conibear et al. 2019). However, these studies were based on glycine peptides, and as glycines have unusual Ramachandran distributions, only some aspects of the neighbor-dependence are accounted for (Kjaergaard and Poulsen 2011). Furthermore, the effect of a PTM may extend further than to its immediate neighbors and previous work has not provided any correction factors for neighboring amino acid residues upon phosphorylation. Recent work on 18 differently modified amino acids measured the random coil CSs of phosphorylated serine, threonine and tyrosine in an Ac-GGXGG-NH_2_ context as well as reported neighboring effect, but only at pH 5.0 (Conibear et al. 2019). This pH is not compatible with most studies on IDPs, which are typically conducted around physiological pH and at, or above, the pI of the phosphates.

At present, the most precise way to extract the inducible effect of either phosphorylation, other PTMs, or non-natural amino acids is to use the chemically unfolded state as internal reference (δ_irc_) to determine the SCSs (Modig et al. 2007). The use of the chemically unfolded state as internal reference does not require a reference library, as each protein can be used as its own internal reference. Thus, this approach has successfully been used in secondary structure analysis of a number of IDPs (Haxholm et al. 2015; Hendus-Altenburger et al. 2016; Kjaergaard et al. 2010), as well as to quantify the effect of multiple phosphorylations on secondary structure and the identification of a stabilizing phospho-motif in an IDP (Hendus-Altenburger et al. 2017). Yet, using the chemically unfolded state requires another round of assignment, homogeneous phosphorylations of all modified sites as well as identical sample conditions for all states, which is rather laborious. Moreover, the extent to which urea biases the CSs is not entirely clear (Elam et al. 2013; Whittington et al. 2005). Thus, peptide derived random coil shifts remain an efficient and accurate approach to examine the locally and globally induced structural changes of these modifications.

Here, we expand the previous dataset of random coil CSs and sequence correction factors recorded on the Ac-QQXQQ-NH_2_ peptide series (Kjaergaard and Poulsen 2011) by including the phosphorylated states of serine, threonine and tyrosine (referred to as pSer, pThr and pTyr). The results have been implemented in an online predictor at www.bio.ku.dk/sbinlab/randomcoil. We have explored the effects at various experimental conditions that are likely to be relevant for phosphorylations in IDPs, specifically the temperature- and pH dependence of the phosphorylated state random coil CSs covering the pKas of the phosphates. Although this dataset was determined with IDPs and the effect of their phosphorylation in mind, it should be equally applicable for folded proteins.

## Materials and methods

Peptides with the sequence Ac-QQXQQ-NH_2_ were purchased from KJ Ross-Petersen ApS (Denmark) and from Schafer N (Denmark), where X was either serine, threonine or tyrosine without or with (pSer, pThr or pTyr) phosphorylation (≥ 95% purity by reversed phase HPLC, identities confirmed by mass spectrometry).

Peptide samples for circular dichroism (CD) analyses were prepared in 20 mM sodium phosphate buffer to a final approximate concentration of 250 µM (pH 6.5, with or without 150 mM NaF). Far-UV CD spectra were recorded from 260 to 190 nm on a Jasco 815 spectropolarimeter in 0.1 cm quartz cuvettes, and with Peltier controlled temperatures set to 5 °C or 35 °C. Each spectrum was recorded at a scan rate of 10 nm/min, band width 1 nm, and a response time of 2 s and averaged over 10 scans. To enable comparison at equal concentrations, especially as the serine, pSer, threonine, and pThr peptides lack absorbance at 280 nm, the signals were normalized using the HT level. Background spectra were recorded identically and subtracted. The final spectra were smoothed using the FFT function in the Jasco software.

NMR samples were prepared by dissolving 2–3 mg of peptide in 500 µL 20 mM sodium phosphate buffer pH 6.5 containing 5% (v/v) D_2_O, 3 mM NaN_3_, and 1 mM DSS. pH was adjusted to 6.5 by the addition of small quantities of HCl or NaOH or in urea as described (Hendus-Altenburger et al. 2017). All NMR spectra were acquired on either a Varian Unity 800 MHz spectrometer equipped with a room temperature probe or a 600 MHz Bruker Avance III HD spectrometer with a cryo-probe. CSs were referenced to internal DSS as previously described (Wishart et al. 1995). For each sample the following spectra were acquired at natural isotope abundance: 1D ^1^H (zgesgp), ^1^H–^15^N HSQC (hsqcetfpf3gpsi), ^1^H–^13^C HSQC (hsqcetgpsisp2.2), 2D TOCSY (Piotto et al. 1992) (mlevgpph19, mixing time 80 ms), 2D ROESY (roesygpph19.2, mixing time 300 ms) and ^1^H^α^–^13^CO HSQC (HACO_hsqcetgpsi) (Kjaergaard et al. 2011). The ^1^H^α^–^13^CO HSQC experiment correlates the H^α^ protons with the carbonyl resonances of the same and the preceding residue. For all peptides, data were recorded at 5 °C, 15 °C, 25 °C, and 35 °C. NMR data were processed using NMRPipe (Delaglio et al. 1995) and analyzed using CCPNMR Analysis (Vranken et al. 2005). The ^3^J_HNHA_ coupling constants were measured by the peak splitting of the H^N^-signals in the 1D ^1^H NMR spectra.

Temperature coefficients were determined at both pH 5.0 and pH 6.5 by least squares fitting of the chemical shift δ to a linear function of temperature T, Eq. (1), where ‘a’ is the temperature coefficient:

1 $$\updelta_{\text{rc}} \left( {\text{T}} \right) = \updelta_{\text{rc}} \left( {25^\circ {\text{C}}} \right) + {\text{a }}\left( {{\text{T}} - 25} \right)$$

Under the assumption that the CS at the center residue of the peptide can be expressed as a linear function of contributions from its neighboring residues, the sequence corrected random coil CSs can be calculated at any temperature using Eq. (2):

2 $$\updelta_{\text{rc}} \left( {\text{T}} \right) = \updelta_{\text{rc}} \left( {25^\circ {\text{C}}} \right) + {\text{a }}\left( {{\text{T}} - 25} \right) + {\text{A}} + {\text{B}} + {\text{C}} + {\text{D}}$$

where A, B, C and D are the correction terms of the subsequent/previous residues. ‘A’ is the sequence correction factor obtained by subtracting the CSs of Q1 of the Ac-QQQQQ-NH_2_ peptide from that of the Ac-QQXQQ-NH_2_ peptides. ‘B’, ‘C’ and ‘D’ correspond to the differences for Q2, Q4, and Q5, respectively.

For the phosphopeptides, NMR titration series from pH 8.0 to 4.0 in steps of ~ 0.5 were recorded. Samples were prepared by dissolving 2–3 mg of peptide in 500 µL 20 mM sodium phosphate buffer, pH 8.0, containing 5% (v/v) D_2_O, 3 mM NaN_3_, and 1 mM DSS, and adjusting the pH stepwise by addition of HCl. For these experiments, the NMR spectra were recorded at 5 °C to minimize exchange of the amide protons with the solvent. The changes in CSs were treated as a linear combination of the CS of a fully protonated and a fully deprotonated species and thus follow Eq. (3):

3 $$\updelta = \updelta_{A} \times \frac{{K_{A} }}{{10^{ - pH} + K_{A} }} + \updelta_{HA} \times \left( {1 - \frac{{K_{A} }}{{10^{ - pH} + K_{A} }}} \right)$$

δ_HA_ and δ_A_ represent the random coil CSs of the fully protonated and fully deprotonated species, respectively. *K*_*a*_ is the acid dissociation constant of the side chain. The CSs were fitted to Eq. (3), where *K*_*a*_ was treated as a global fitting parameter. The protonation/deprotonation of the N- and C-termini could be neglected due to N-terminal acetylation and C-terminal amidation.

As is evident from the expressions above, calculation of the random coil shifts is computationally efficient. The parameters are stored in simple lookup tables in a Javascript program, extending the previous implementation (Kjaergaard et al. 2011) with three additional residue types. To provide support for calculating neighbor-dependent correction factors at all pH values, we linearly interpolated between the recorded values in our range (corresponding to the lines connecting the observations in Fig. 2). The script supports calculation for entire sequences at once. Since there is no established single-letter notation for the phosphorylated amino acids, we allow mixing of single-letter and multi-letter amino code specification by surrounding the latter by parentheses. For instance, the string “A(Ala)(pSer)” is interpreted as two alanines followed by a phosphorylated serine. The phosphorylated amino acid can be specified using several common conventions, i.e. either pS, pT, pY, pSer, pThr, pTyr, or Sep, Tpo, Ptr. The on-line predictor can be found at: www.bio.ku.dk/sbinlab/randomcoil.

To test the performance of the new predictor, the chemical shifts of six phosphorylated proteins were extracted from the BMRB database. These include the sodium proton exchanger 1 (NHE1) (BMRB 26755 and 27812) (Hendus-Altenburger et al. 2017; Hendus-Altenburger et al. 2016), the kinase inducible transactivation domain (KID) (BMRB 6784 and 6788) (Radhakrishnan et al. 1998), the transcriptional regulator protein Ash1 (BMRB 26719 and 26720) (Martin et al. 2016), the disordered cytosolic domain CD79a of the B cell receptor (BMRB 19644 and 19648) (Rosenlow et al. 2014), the regulatory region of the cystic fibrosis transmembrane conductance regulator (CFTR) (BMRB 15336 and 15340) (Baker et al. 2007) and the transcriptional activation domain of the transcription factor Elk-1 (BMRB 26762 and 26786) (Mylona et al. 2016).

## Results and discussion

The change in NMR CSs upon PTMs of proteins is due to the changed local chemical environment but can in addition be caused by accompanying structural rearrangements induced by the PTM. While the chemical effect is not expected to reach further than 2–3 residues on either side of the modified residue in the random coil state, structural rearrangements or changes in the conformational ensembles of IDPs can have long-range effects (Hendus-Altenburger et al. 2017). In order to allow for secondary structure analysis in the presence of phosphorylation we extended the previously published Ac-QQXQQ-NH_2_ peptide random coil CS database to include those for phosphorylated serine (pSer), threonine (pThr) and tyrosine (pTyr) residues. Furthermore, the random coil CS were extracted for various temperatures and at pH values ranging from pH 4.0 to 8.0 to cover the pKas of the phosphates.

**Fig. 1:**
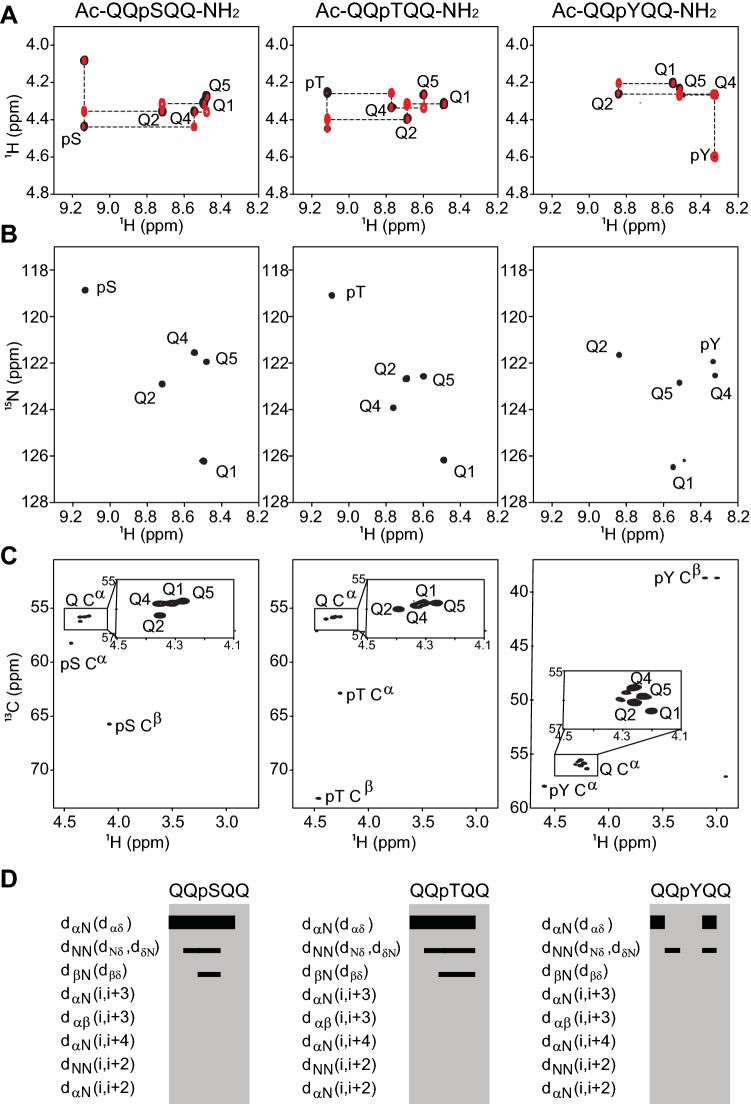
NMR spectra of the Ac-QQXQQ-NH_2_ phosphopeptides. The CSs were assigned from combining **a** 2D TOCSY (black) and ROESY (red), **b**^1^H–^15^N HSQC, **c**^1^H–^13^C HSQC spectra. **d** ROE patterns for Ac-QQpXQQ-NH_2_ phosphopeptides (pH 6.5, 5 °C). The intensities of the ROEs are indicated by the height of the black bars

Assignments and CSs for serine, threonine and tyrosine in the Ac-QQXQQ-NH_2_ context were readily transferred from the previous study (Kjaergaard and Poulsen 2011) and the CSs of their phosphorylated states were assigned by a combination of TOCSY and ROESY spectra (Fig. 1a). The random coil CSs of C^α^, C^β^, C′, N, H^N^ and H^α^ were determined for each phosphopeptide at different temperatures (5–35 °C) and in the physiological pH range from pH 4.0–8.0, by recording the following spectra: ^1^H–^15^N HSQC, ^1^H–^13^C HSQC, 2D TOCSY, and ^1^H^α^–^13^CO HSQC. The CSs of the phosphorylated residues of the peptides at pH 6.5 and 5 °C are tabulated in Table 1.

**Table 1:** Random coil CSs at pH 6.5 and 5°C

	δ_RC_ (ppm)					
	C^α^	C^β^	C′	N	H^N^	H^α^
Ser	58.60	63.71	174.80	117.63	8.59	4.44
Thr	62.15	69.88	174.65	116.38	8.41	4.34
Tyr	57.97	38.65	175.77	121.79	8.40	4.59
pSer	58.24	65.73	174.77	118.87	9.13	4.44
pThr	62.99	72.45	174.57	119.10	9.09	4.25
pTyr	57.99	38.70	175.79	121.94	8.34	4.60

The amide peaks were well resolved in the ^1^H–^15^N HSQC spectra of all three phosphorylated peptides (Fig. 1b) and their CSs were readily assigned as indicated in the ^1^H–^13^C–HSQC spectra (Fig. 1c). For pSer and pThr, strong downfield shifts of the backbone amides were observed compared to the unphosphorylated counterparts. In contrast, tyrosine phosphorylation did not induce a similar large downfield shift of the modified residue, likely due to the more distal position of the modified hydroxyl group in the side chain relative to the backbone amide (Bienkiewicz and Lumb 1999; Theillet et al. 2012) (Table 1). These are important observations, as in several cases (transient) hydrogen bonds between the phospho-group and the amide of the same or neighboring residues were observed upon phosphorylation (Du et al. 2005; Ramelot and Nicholson 2001; Kang et al. 2010). We note that Q2 of the pTyr peptide showed a strong down-field shift suggesting this residue to be more affected than the tyrosine amide itself, which indicates that the CS of the residue prior to a pTyr can be used diagnostically to identify phosphorylation of tyrosine residues by NMR.

Within error, the random coil shifts for pTyr as well as the shift of its neighboring glutamines were identical to those of the non-phosphorylated peptides, testifying to the random coil nature of the phosphorylated peptide. However, for pSer and pThr, the shifts deviated from those of the non-phosphorylated peptides and together with the downfield shift of the amide, this could indicate structure formation. Thus, to address if the phosphates in these peptides induce structure, we recorded far-UV CD spectra of the phosphorylated as well as non-phosphorylated peptides at different temperatures (5 °C and 35 °C) and in the absence and presence of 150 mM NaF (SI Fig. S1). The peptides were all in a random coil state as judged by the negative ellipticity at 198 nm and the slight positive signal at 215 nm. Besides a more pronounced negative ellipticity at 198 nm for the phosphorylated peptides, indicating slightly more extended structure, phosphorylation did not change the CD spectra, neither did the presence of 150 mM NaF. At 35 °C, we observed a minor change in the CD profile towards a slight redistribution away from polyproline II structure, as observed previously for IDPs (Kjaergaard et al. 2010). Finally, we compared the ^1^H,^13^C-HSQC spectra recorded on Ac-QQpSQQ-NH_2_ and Ac-QQpTQQ-NH_2_ in the absence and presence of 150 mM NaCl (SI Fig. S2), which showed the C^α^, C^β^, and H^α^ CSs to be similar. Thus, the presence of salt at physiological concentrations does not change the conformational ensemble of these peptides.

An overlay of the ^15^N-HSQC spectra of the phosphorylated and non-phosphorylated peptides showed that the glutamine side chain resonances did not readily superimpose (SI Fig. S3), indicating that the phosphates changed the chemical environment of these and/or induced structure. Therefore, to further substantiate the random coil nature of the phosphorylated peptides, we analyzed ROESY spectra for connectivity beyond those of sequential origin. All phosphorylated peptides showed stronger H^α^–H^N^ (i, i + 1) inter-residue cross-peaks, and weaker H^N^–H^N^ (i, i + 1) cross peaks (Fig. 1d), showing that the phosphorylated peptides are indeed random coil and have no secondary structure related interactions (Bienkiewicz and Lumb 1999; Dyson and Wright 1991). We repeated the ROESY spectra in the presence of 150 mM NaCl and 8 M urea. These changes did not alter the peak intensity patterns and thus the ensembles remained similar (SI Fig. S4).

**Fig. 2:**
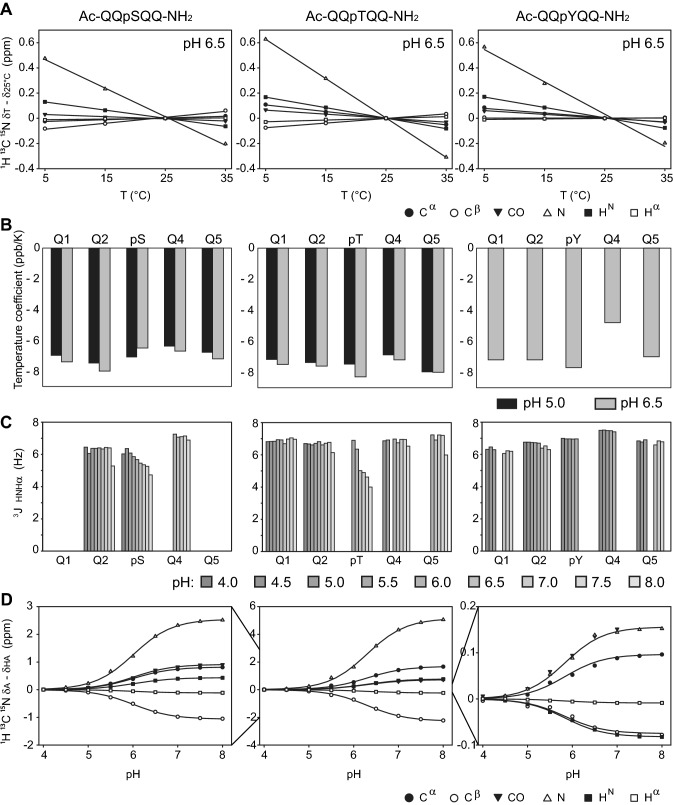
Temperature and pH dependence of the random coil chemical shifts of the Ac-QQXQQ-NH_2_ phosphopeptides. **a** Temperature dependence of the chemical shifts of pSer, pThr and pTyr recorded at pH 6.5. Random coil chemical shifts assigned at 25 °C were subtracted from the shifts recorded at the other temperatures to allow the different nuclei to be presented together. Lines represent the best linear fit to the change in chemical shifts, from which the temperature coefficients were extracted. **b** Temperature coefficients of the amide protons of the phosphopeptides at pH 6.5 (pSer, pThr, pTyr) and pH 5.0 (pSer, pThr). **c**^3^J_HNHα_ coupling constants as a function of pH for pSer, pThr and pTyr. Missing bars are either due to decoupling or severe peak overlap. **d** The pH dependence of the chemical shifts of pSer, pThr, and pTyr determined in a titration series from pH 4.0 to 8.0 at 5 °C

The temperature dependence of the backbone CSs was next determined from a series of NMR spectra recorded at 5 °C, 15 °C, 25 °C and 35 °C. The CSs changed linearly with temperature (Fig. 2) and the CSs and temperature coefficients for each residue type and nuclei were readily extracted (Table 1). Temperature coefficients for the amide protons can serve as indicators for hydrogen bond formation (Cierpicki and Otlewski 2001), although this has been debated (Tholey et al. 1999; Kim et al. 2011; Rani and Mallajosyula 2017). None of the three phosphopeptides revealed amide temperature coefficients larger (more positive) than − 4.6 ppb/K neither at pH 6.5 or at pH 5.0 (Fig. 2b), which suggests that phosphorylation may not be enough to form a persistent hydrogen bond with the backbone amide. However, measurements of the ^3^J_HNHA_ coupling constants over a pH range from 4.0 to 8.0 showed that those of the phosphorylated serine and threonine, but not those of the tyrosine or the glutamines, were pH dependent. At low pH, the ^3^J_HNHA_ coupling constants had random coil values (Shen et al. 2018), but decreased and reached 4.7 Hz (pSer) and 4.0 Hz (pThr), respectively, at pH 8.0 (Fig. 2c). This either suggests that there is steric exclusion from the presence of the phosphoryl groups or that a (transient) hydrogen bond is formed in the deprotonated state. The latter conclusion is in line with the literature (Du et al. 2005; Tholey et al. 1999; Kim et al. 2011; Mandell et al. 2007; Lee et al. 2008) and suggest that a (transient) hydrogen bond may be part of the high pH-random coil state of pSer and pThr. The remaining context, constituted by the four glutamines, were random coil in the entire temperature- and pH range analyzed and in the presence of salt. Thus, we conclude that these peptides are suitable representations of the phosphorylated random coil state.

The random coil CSs of the backbone change with the protonation state of the side chains and with the potential hydrogen bond formation at high pH, so to allow the random coil CSs to be used in a range of pH values, pH titrations were carried out for peptides with pSer, pThr and pTyr (Fig. 2d). The CSs at different pH values were then fitted to Eq. (3), which describes the observed CSs as a linear combination of the CSs for the fully protonated and the fully deprotonated states, respectively, as a function of pH (Table 2). From these fits, we obtained pKa values for pSer, pThr and pTyr, of 6.01, 6.30 and 5.83, respectively; similar, but not identical, to the previously determined values for Ac-GGXGG-NH_2_ peptides (pKa 5.96, 6.30, 5.96) (Bienkiewicz and Lumb 1999) and to those in a GGXA context (pKa 6.1, 6.1, 5.9) (Hoffmann et al. 1994). The largest differences in CSs between the protonated and deprotonated state were those observed for the amide nitrogen, and strongest for pThr (ΔδN = 5.16), followed by pSer (ΔδN = 2.55), and only small differences were observed for pTyr (ΔδN = 0.15) (Fig. 2d; Table 2). Importantly, the amplitude of the change in CS occurring by altering the protonation state is in the same range—or even larger—as for transient structure formation in IDPs. Thus, these effects may falsely be interpreted as such, unless the random coil CSs used in the calculations are matched to the pH of the NMR sample. However, the pKa value of individual phosphorylation sites in the protein context will depend on several other factors, including the presence of charges or polar residues in the neighboring sequence, additional phosphorylation sites (Tamiola et al. 2018), as well as local or, in the case of a folded protein, the global protein conformation (Buckingham 1960; Kukic et al. 2013; Wishart 2011; Tomlinson et al. 2010). In these cases, care should be taken in interpreting effects of phosphorylation, and a pKa determination of the different sites in urea, which may also provide the intrinsic random coils shifts, can be a solution.

**Table 2:** Random coil CSs of the fully protonated and fully deprotonated phosphorylated residues at 5 °C

	Ka pKa		C^α^	C^β^	C′	N	H^N^	H^α^
pSer	9.76 × 10^−7^ 6.01	δ_HA_	57.61	66.53	174.06	116.94	8.81	4.52
		δ_A_	58.43	65.46	174.98	119.49	9.24	4.41
		δ_A_ − δ_HA_	0.82	− 1.07	0.92	2.55	0.43	− 0.11
pThr	5.00 × 10^−7^ 6.30	δ_HA_	61.83	73.99	174.06	115.94	8.62	4.41
		δ_A_	63.52	71.72	174.79	121.10	9.40	4.17
		δ_A_ − δ_HA_	1.69	− 2.27	0.73	5.16	0.79	− 0.24
pTyr	1.47 × 10^−6^ 5.83	δ_HA_	57.91	38.76	175.61	121.80	8.40	4.60
		δ_A_	58.00	38.69	175.79	121.95	8.32	4.59
		δ_A_ − δ_HA_	0.09	− 0.07	0.18	0.15	− 0.08	− 0.01

## Sequence correction

The effect of phosphorylation on the CSs may extend beyond the nearest neighbor residue and sequence correction factors were therefore extracted for residue X by subtracting the CSs of the Ac-QQQQQ-NH_2_ peptide from those of the Ac-QQXQQ-NH_2_ peptide (Table 3) (Schwarzinger et al. 2001). Generally, the sequence correction factors for carbon nuclei are larger for residues further away on the N-terminal side, whereas for the amide, they are larger on the C-terminal side. Also, the sign of the effect is opposite for pTyr compared to pThr and pSer, likely due to ring current effects. The largest effects are in the order of 0.35–0.6 ppm, which means that if not accounted for, this can lead to an over/underestimation of the helical content of the region by up to 20% [assuming 100% helicity will result in SCS of 2.8 ppm (Fedyukina et al. 2010)], a number that is close to the typical population of helicity in IDPs (Hendus-Altenburger et al. 2017; Forman-Kay and Mittag 2013). Importantly, this also suggests that we may miss the identification of direct structural effects of phosphorylation in IDPs if we do not take the sequence effect into consideration. Finally, as the titration of the phosphate may also change the sequence correction factors, we extracted these over the pH range from pH 4.0 to 8.0 (SI Table 1).

**Table 3:** Glutamine derived sequence correction factors at pH 6.5, 5 °C

	C^α^				C^β^				C′			
	A	B	C	D	A	B	C	D	A	B	C	D
pSer	− 0.30	0.18	− 0.16	− 0.01	0.01	0.08	− 0.18	− 0.06	− 0.14	0.25	0.00	0.12
pThr	− 0.30	− 0.04	− 0.10	0.06	0.08	0.21	0.00	− 0.03	− 0.37	0.07	− 0.13	0.10
pTyr	0.26	0.04	− 0.40	0.13	− 0.15	− 0.51	0.08	− 0.02	− 0.03	− 0.25	− 0.61	0.05

	N				H^N^				H^α^			
	A	B	C	D	A	B	C	D	A	B	C	D
pSer	− 0.02	0.72	− 0.76	− 0.82	− 0.01	− 0.01	− 0.12	− 0.17	0.03	0.03	0.03	− 0.03
pThr	− 0.05	0.48	1.62	− 0.19	− 0.02	− 0.04	0.09	− 0.06	0.03	0.07	0.01	− 0.04
pTyr	0.25	− 0.53	0.23	0.08	0.04	0.11	− 0.34	− 0.14	− 0.08	− 0.06	− 0.05	− 0.07

‘A’ is the sequence correction factor obtained by subtracting the CS of Q1 of the Ac-QQQQQ-NH_2_ peptide from that of the Ac-QQXQQ-NH_2_ peptides. ‘B’, ‘C’ and ‘D’ correspond to the differences for Q2, Q4, and Q5, respectively

## Revisiting the CS analyses of phosphorylated IDPs

Since the obtained data allows for a more direct comparison of the effect of phosphorylation on the IDP ensemble from CS analyses only, we revisited previous published CS data sets and identified a set of phosphorylated IDPs published in the BMRB (Table 4). The following proteins were revisited: (i) the intrinsically disordered distal tail of NHE1 phosphorylated at five serine residues and one threonine by the MAPK kinase ERK2 (Hendus-Altenburger et al. 2017; Hendus-Altenburger et al. 2016), (ii) the disordered KID domain of the transcription factor CREB phosphorylated at one serine by protein kinase A (PKA) (Radhakrishnan et al. 1998), (iii) the transcriptional regulator Ash1 phosphorylated at eight serine and two threonine residues by cyclin A/Cdk2 (Martin et al. 2016), (iv) the disordered cytosolic domain CD79a of the B-cell receptor phosphorylated at four tyrosine residues by the Src family kinase Fyn (Rosenlow et al. 2014), (v) the regulatory region of CFTR fully phosphorylated at eight serine residues by PKA (Baker et al. 2007) and (vi) the transcriptional activation domain of the transcription factor Elk-1 phosphorylated at five threonine and three serine residues by ERK2 (Mylona et al. 2016). Besides NHE1, which had been previously analyzed using intrinsic reference coil values, the five additional proteins were chosen as they are phosphorylated by different kinases and thus have different substrate motifs surrounding the phosphorylation sites, as well as represent several examples of pSer, pThr and pTyr. Furthermore, we chose proteins with assignments around pH 7.0, which is close to the physiological range and commonly used for IDP studies. In total, this provided us with the possibility of examining the effects from four different kinases and phosphorylations of 25 serine-, eight threonine-, and four tyrosine sites. This allowed for extraction of some general observations.

**Table 4:** CSs of phosphorylated proteins available in the BMRB and used in this study

Protein	Phosphorylation sites (kinase)	BMRB
Na^+^/H^+^ exchanger 1 (NHE1), disordered distal tail	Ser693, Ser723, Ser726, Ser771, Thr779, Ser785 (MAP kinase ERK2)	26755 27812 (Hendus-Altenburger et al. 2017; Hendus-Altenburger et al. 2016)
Kinase inducible transactivation (KID) domain of the transcription factor CREB	Ser133 (Protein kinase A, PKA)	6784 6788 (Radhakrishnan et al. 1998)
Regulatory region of the cystic fibrosis transmembrane conductance regulator (CFTR)	Ser660, Ser700, Ser712, Ser737, Ser753, Ser768, Ser795, Ser813 (Protein kinase A, PKA)	15336 15340 (Baker et al. 2007)
Transcriptional regulator protein Ash1	Ser424, Ser426, Thr429, Ser442, Thr450, Ser452, Ser455, Ser465, Ser469, Ser490 (cyclin A/Cdk2)	26719 26720 (Martin et al. 2016)
Activation domain of the transcription factor Elk-1	Thr337, Thr354, Thr364, Thr369, Ser384, Ser390, Thr418, Ser423 (MAP kinase ERK2)	26762 26786 (Mylona et al. 2016)
Disordered cytosolic domain CD79a of the B-cell receptor	Tyr182, Tyr188, Tyr199, Tyr210 (Src family kinase Fyn)	19644 19648 (Rosenlow et al. 2014)

**Fig. 3:**
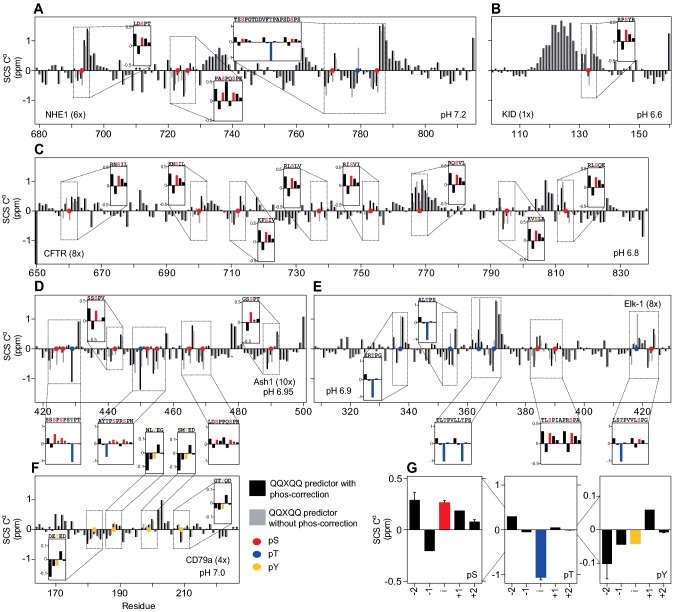
Difference in secondary chemical shift prediction with and without the reference chemical shifts and correction factors for phosphorylated residues. Secondary C^α^ chemical shifts (SCS C^α^) from the predictor lacking the reference chemical shifts and correction factors for phosphorylated residues (grey) and with these included in the updated predictor (black) of the following phosphorylated proteins **a** NHE1, **b** KID domain, **c** R-region of CFTR, **d** Ash1, **e** Elk1, transactivation domain and **f** CD79a. Subtraction of the SCS of the previous predictor from the SCS of the updated predictor (SCS_new_ − SCS_old_) are shown for each phosphorylated region in the highlighted boxes. **g** Difference for pSer, pThr and pTyr, averaged over all analyzed sites. Colored bars, dots and letters indicate the phosphorylated residues. Black stars indicate unassigned residues

For each phosphorylated protein, we calculated the random coil CS of the C^α^s with and without the new corrections for phosphorylations and plotted these side by side on a per residues basis (Fig. 3a–e). For the residues surrounding the phosphorylation sites we calculated the difference (δ_RCnon-phos_ − δ_RCphos_, see inserts in Fig. 3a–e) and averaged these for each of the three residue types (Fig. 3g). Generally, we observed that the differences in random coil shifts were independent on the neighboring residues or the kinase used, as expected. For a phosphorylated serine, the use of a unphosphorylated random coil set underestimates the SCS of the C^α^ of the i − 2 position with 0.3 ppm, overestimates it with 0.15 ppm at the i − 1 position, and with 0.15 ppm at the pSer, with minor overestimations on the i + 1 and i + 2 positions. For the phosphorylated threonine residues, the effects are much stronger. Similar underestimations of 0.3 ppm at the i − 2 position are seen, but with overestimations of as much as 1 ppm at the pThr position (i). Smaller underestimations are seen on the i + 1 and i + 2 positions. Much smaller effects were again seen for the phosphorylated tyrosine, with highly similar effects independent of the sequence.

**Fig. 4:**
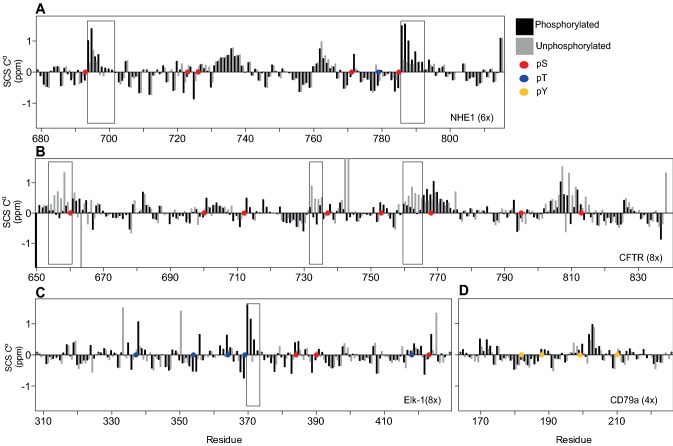
Effect of phosphorylation on secondary structure in a selected set of IDPs. Secondary C^α^ chemical shifts (SCS C^α^) for the unphosphorylated and phosphorylated states are compared for **a** the disordered distal tail of NHE1, **b** R-region of CFTR, **c** Elk-1, and **d** CD79a. Boxes indicate regions with structural modulation. Colored dots indicate the phosphorylated residues (red/blue/yellow for pSer/pThr/pTyr respectively). Black stars indicate unassigned residues

We then translated these effects into how stabilization and destabilization of transient structures by phosphorylations would potentially be masked or modulated by the absence of a proper random coil CS data set. For this, we examined the CSs of a set of phosphorylated proteins and compared the SCSs in the unphosphorylated state with those of the phosphorylated state using the implemented random coil sets (Fig. 4). For NHE1, the six times ERK2-phosphorylated state had previously been analyzed using intrinsic random coil values, where the CSs of the phosphorylated state were assigned in urea (Hendus-Altenburger et al. 2017). It was evident that phosphorylations induced helicity at two out of six sites, which could be linked to the presence of arginine residues positioned in i + 5 positions (Hendus-Altenburger et al. 2017). The SCSs derived using the predictor accounting for phosphorylation agree very well with those derived from the urea unfolded state, detecting helix stabilization by pSer693 and pSer785 with no effects induced by the other four phosphorylations. However, omitting phosphorylation correction, this effect was underestimated roughly fourfold (Fig. 4a). For CFTR it was previously suggested that there was a global decrease in helicity upon phosphorylation, with stabilization of the helix by pSer768, while pSer700, pSer737 and pSer813 all destabilized helices. Using the new predictor, three regions with a decrease in helicity can be observed, all located N-terminal to the phosphorylation sites, i.e. N-terminal to pSer660, pSer737 and pSer768. Also, a minor stabilization at pSer768 between i − 2 until i + 1 is detected. However, no significant effect is seen for pS700 and more random changes are observed for pS813 (Fig. 4b). This highlights the importance of separating structural from chemical effects, as omitting this can lead to over/underestimation of structural modulation by phosphorylation. For Elk-1, most phosphorylations had no significant effect on the secondary structure except for pSer369, where induction of helicity was observed (Fig. 4c). No significant effect of tyrosine phosphorylation on the secondary structure was seen for CD79a (Fig. 4d).

Taken together, the use of the new predictor of random coil CS values for phosphorylated proteins allowed for more accurate detection of the transient secondary structures of phosphoproteins. The effects of phosphorylation could now be directly separated from the chemical effect and enabled quantification of the structure modulating effects of phosphorylation. Generally, a phosphorylation N-terminal to transient helicity stabilized the helical structure, and when the phosphorylation site was positioned C-terminal to transient structure, helicity was destabilized, in agreement with previous observations (Andrew et al. 2002). Further, the new predictor allows for pH corrected CS predictions, which is critical as phosphate titrates in the physiological pH range with considerable effects on the carbon CSs, eliminating spurious spikes in the secondary CSs. The large effect of phosphorylation on threonine CSs combined with its strong pH sensitivity, warrants extra care in interpreting structural effects from threonine phosphorylation in general.

Extraction of local structure from CSs has been possible for 50 years (Markley et al. 1967) and the random coil CS databases and peptide-derived libraries continue to improve both in accuracy and precision of the correction factors for sequence and sample conditions like temperature and pH (Kjaergaard et al. 2011; Kjaergaard and Poulsen 2011; Schwarzinger et al. 2001). With the inclusion of a full set of random coil shifts for phosphorylated side chains in proteins and their pH and temperature dependence covering the range of the pKa values of the phosphates, we can more reliably analyze and decompose the effects of phosphorylations on the structural ensemble.

## Electronic supplementary material

Below is the link to the electronic supplementary material.

Supplementary material 1—Far-UV CD spectra recorded on (A) Ac-QQSQQ-NH_2_ (black) and Ac-QQpSQQ-NH_2_ (red) and (B) Ac-QQTQQ-NH_2_ (black) and Ac-QQpTQQ-NH_2_ (red) in 20 mM sodium phosphate, pH 6.5 at 5°C and 35°C and in the absence or presence of 150 mM NaF. (EPS 1144 kb)

Supplementary material 2—Overlay of ^1^H,^13^C-HSQC spectra of (A) Ac-QQpSQQ-NH_2_, (B) Ac-QQpTQQ-NH_2_ (C) Ac-QQpYQQ-NH_2_ recorded in 20 mM sodium phosphate, pH 6.5, 5°C in the absence (black) and presence (red) of 150 mM NaCl. (EPS 989 kb)

Supplementary material 3—Overlay of full ^1^H,^15^N-HSQC spectra of (A) Ac-QQSQQ-NH_2_ (black) and Ac-QQpSQQ-NH_2_ (red), (B) Ac-QQTQQ-NH_2_ (black) and Ac-QQpTQQ-NH_2_ (red), (C) Ac-QQYQQ-NH_2_ (black) and Ac-QQpYQQ-NH_2_ (red) recorded in 20 mM sodium phosphate, pH 6.5 at 5°C. Stars indicate folded peaks reporting on the C-terminal amidation (NH_2_). (EPS 1184 kb)

Supplementary material 4—ROE intensities for Ac-QQpSQQ-NH_2_, Ac-QQpTQQ-NH_2_ and Ac-QQpYQQ-NH_2_ in (A) 150 mM NaCl or (B) in 8 M urea. (EPS 809 kb)

Supplementary material 5 (DOCX 27 kb)

## Data Availability

The online predictor of random coil chemical shifts for unphosphorylated and phosphorylated sequences is available at www.bio.ku.dk/sbinlab/randomcoil.
