# Supplementary material for: Random coil chemical shifts for serine, threonine and tyrosine phosphorylation over a broad pH range
Source: J Biomol NMR. 2019 Oct 9;73(12):713–25. doi: 10.1007/s10858-019-00283-z (PMC6875518; doi:10.1007/s10858-019-00283-z)
Supplement: Supplementary file 5 — Supplementary material 5 (DOCX 27 kb) [file 10858_2019_283_MOESM5_ESM.docx]

**Table S1. Temperature coefficients of pSer, pThr and pTyr at pH 6.5 and 5.0, 20 mM sodium phosphate.**

| Temperature coefficient (ppb/K) | | | | | | |
| --- | --- | --- | --- | --- | --- | --- |
| pH 5.0 | C^α^ | C^β^ | C’ | N | H^N^ | H^α^ |
| pSer | 0.7 | 4.0 | - | -22.0 | -7.1 | 0.5 |
| pThr | -0.5 | 0.2 | - | -20.2 | -7.5 | 0.3 |
| pH 6.5 | C^α^ | C^β^ | C’ | N | H^N^ | H^α^ |
| pSer | 1.4 | 4.7 | -1.7 | -22.7 | -6.5 | 0.5 |
| pThr | -5.3 | 3.7 | -3.0 | -31.3 | -8.3 | 1.4 |
| pTyr | -3.7 | 0.0 | -3.0 | -25.6 | -8.3 | 0.4 |

**Table S2. Sequence correction factors at different pH values.**

|  |  | C^α^ |  |  |  | C^β^ |  |  |  | C’ |  |  |  |
| --- | --- | --- | --- | --- | --- | --- | --- | --- | --- | --- | --- | --- | --- |
|  |  | A | B | C | D | A | B | C | D | A | B | C | D |
| pS | 4.0 | -0.18 | 0.28 | -0.10 | 0.00 | -0.05 | 0.07 | -0.08 | -0.08 | 0.05 | 0.27 | -0.06 | 0.04 |
|  | 4.5 | -0.19 | 0.27 | -0.11 | 0.00 | -0.04 | 0.07 | -0.10 | -0.08 | 0.02 | 0.23 | 0.00 | 0.09 |
|  | 5.0 | -0.23 | 0.25 | -0.12 | -0.01 | -0.06 | 0.05 | -0.08 | -0.10 | 0.02 | 0.26 | -0.01 | 0.09 |
|  | 5.5 | -0.22 | 0.24 | -0.12 | 0.00 | -0.04 | 0.07 | -0.13 | -0.07 | -0.04 | 0.25 | -0.12 | 0.06 |
|  | 6.0 | -0.26 | 0.21 | -0.14 | -0.01 | -0.04 | 0.05 | -0.16 | -0.11 | -0.05 | 0.25 | 0.01 | 0.09 |
|  | 6.5 | -0.30 | 0.18 | -0.16 | -0.01 | 0.01 | 0.08 | -0.18 | -0.06 | -0.14 | 0.25 | 0.00 | 0.12 |
|  | 7.0 | -0.33 | 0.13 | -0.16 | -0.02 | -0.01 | 0.09 | -0.19 | -0.05 | -0.25 | 0.27 | 0.00 | 0.18 |
|  | 7.5 | -0.33 | 0.15 | -0.17 | -0.01 | 0.01 | 0.10 | -0.19 | -0.06 | -0.16 | 0.26 | 0.05 | 0.18 |
|  | 8.0 | -0.33 | 0.14 | -0.17 | -0.01 | 0.02 | 0.10 | -0.20 | -0.05 | -0.23 | 0.25 | 0.01 | 0.17 |
| pT | 4.0 | -0.22 | -0.03 | -0.08 | 0.05 | 0.04 | 0.12 | 0.04 | -0.11 | -0.18 | 0.27 | -0.14 | 0.02 |
|  | 4.5 | -0.23 | -0.03 | -0.08 | 0.05 | 0.04 | 0.13 | 0.07 | -0.14 | -0.19 | 0.26 | -0.15 | 0.06 |
|  | 5.0 | -0.25 | -0.04 | -0.09 | 0.04 | 0.04 | 0.13 | 0.07 | -0.10 | -0.21 | 0.26 | -0.14 | 0.09 |
|  | 5.5 | -0.25 | -0.03 | -0.09 | 0.05 | 0.05 | 0.09 | 0.01 | -0.14 | -0.22 | 0.23 | -0.15 | 0.06 |
|  | 6.0 | -0.27 | -0.04 | -0.10 | 0.05 | 0.06 | 0.10 | 0.05 | -0.10 | -0.27 | 0.20 | -0.14 | 0.12 |
|  | 6.5 | -0.30 | -0.04 | -0.10 | 0.06 | 0.08 | 0.21 | 0.00 | -0.03 | -0.37 | 0.07 | -0.13 | 0.10 |
|  | 7.0 | -0.35 | -0.05 | -0.13 | 0.06 | 0.10 | 0.29 | -0.04 | -0.05 | -0.39 | 0.01 | -0.13 | 0.13 |
|  | 7.5 | -0.35 | -0.06 | -0.12 | 0.06 | 0.11 | 0.33 | -0.06 | -0.05 | -0.41 | 0.01 | -0.11 | 0.13 |
|  | 8.0 | -0.36 | -0.06 | -0.12 | 0.06 | 0.11 | 0.34 | -0.07 | -0.05 | -0.42 | -0.02 | -0.13 | -0.26 |
| pY | 4.0 | 0.18 | 0.00 | -0.44 | 0.15 | -0.06 | -0.34 | 0.14 | -0.05 | -0.10 | -0.33 | -0.71 | 0.07 |
|  | 4.5 | 0.18 | 0.00 | -0.44 | 0.15 | -0.07 | -0.34 | 0.13 | -0.06 | -0.05 | -0.35 | -0.67 | 0.03 |
|  | 5.0 | 0.19 | 0.00 | -0.44 | 0.14 | -0.08 | -0.37 | 0.13 | -0.05 | -0.04 | -0.38 | -0.54 | 0.05 |
|  | 5.5 | 0.22 | 0.02 | -0.42 | 0.14 | -0.11 | -0.44 | 0.12 | -0.03 | -0.07 | -0.30 | -0.61 | 0.04 |
|  | 6.0 | 0.24 | 0.03 | -0.41 | 0.14 | -0.13 | -0.44 | 0.10 | -0.03 | -0.05 | -0.35 | -0.58 | 0.03 |
|  | 6.5 | 0.26 | 0.04 | -0.40 | 0.13 | -0.15 | -0.51 | 0.08 | -0.02 | -0.03 | -0.25 | -0.61 | 0.05 |
|  | 7.0 | 0.28 | 0.05 | -0.40 | 0.13 | -0.17 | -0.54 | 0.07 | -0.01 | -0.01 | -0.25 | -0.50 | 0.03 |
|  | 7.5 | 0.29 | 0.05 | -0.39 | 0.13 | -0.17 | -0.55 | 0.07 | -0.01 | 0.00 | -0.26 | -0.53 | 0.18 |
|  | 8.0 | 0.29 | 0.05 | -0.39 | 0.13 | -0.19 | -0.57 | 0.06 | -0.01 | 0.00^#^ | -0.26^#^ | -0.55^#^ | 0.20^#^ |
|  |  | N |  |  |  | H^N^ |  |  |  | H^α^ |  |  |  |
|  |  | A | B | C | D | A | B | C | D | A | B | C | D |
| pS | 4.0 | -0.10 | 0.37 | -0.71 | -0.64 | -0.01 | 0.03 | -0.17 | -0.16 | 0.02 | 0.03 | 0.05 | -0.02 |
|  | 4.5 | -0.10 | 0.39 | -0.71 | -0.65 | -0.01 | 0.03 | -0.16 | -0.16 | 0.02 | 0.03 | 0.05 | -0.02 |
|  | 5.0 | -0.09 | 0.43 | -0.72 | -0.67 | -0.01 | 0.02 | -0.16 | -0.17 | 0.02 | 0.02 | 0.04 | -0.02 |
|  | 5.5 | -0.08 | 0.48 | -0.73 | -0.70 | -0.01 | 0.01 | -0.15 | -0.17 | 0.02 | 0.03 | 0.04 | -0.02 |
|  | 6.0 | -0.06 | 0.59 | -0.76 | -0.77 | -0.01 | 0.00 | -0.14 | -0.17 | 0.03 | 0.03 | 0.03 | -0.02 |
|  | 6.5 | -0.02 | 0.72 | -0.76 | -0.82 | -0.01 | -0.01 | -0.12 | -0.17 | 0.03 | 0.03 | 0.03 | -0.03 |
|  | 7.0 | -0.02 | 0.77 | -0.79 | -0.88 | -0.01 | -0.02 | -0.11 | -0.17 | 0.03 | 0.04 | 0.03 | -0.02 |
|  | 7.5 | -0.01 | 0.79 | -0.79 | -0.89 | -0.01 | -0.02 | -0.11 | -0.17 | 0.04 | 0.03 | 0.03 | -0.03 |
|  | 8.0 | -0.01 | 0.80 | -0.79 | -0.89 | -0.01 | -0.03 | -0.11 | -0.17 | 0.04 | 0.03 | 0.03 | -0.03 |
| pT | 4.0 | -0.14 | 0.33 | 0.42 | -0.08 | -0.03 | 0.00 | -0.09 | -0.02 | 0.02 | 0.10 | 0.04 | -0.02 |
|  | 4.5 | -0.13 | 0.33 | 0.45 | -0.09 | -0.03 | 0.00 | -0.08 | -0.02 | 0.02 | 0.10 | 0.04 | -0.02 |
|  | 5.0 | -0.13 | 0.35 | 0.51 | -0.10 | -0.03 | 0.00 | -0.08 | -0.02 | 0.01 | 0.10 | 0.03 | -0.03 |
|  | 5.5 | -0.11 | 0.37 | 0.77 | -0.12 | -0.02 | -0.01 | -0.03 | -0.03 | 0.02 | 0.09 | 0.03 | -0.03 |
|  | 6.0 | -0.10 | 0.41 | 1.04 | -0.15 | -0.02 | -0.02 | 0.01 | -0.04 | 0.02 | 0.08 | 0.02 | -0.03 |
|  | 6.5 | -0.05 | 0.48 | 1.62 | -0.19 | -0.02 | -0.04 | 0.09 | -0.06 | 0.03 | 0.07 | 0.01 | -0.04 |
|  | 7.0 | -0.04 | 0.53 | 2.05 | -0.24 | -0.01 | -0.06 | 0.16 | -0.07 | 0.03 | 0.06 | 0.00 | -0.04 |
|  | 7.5 | -0.03 | 0.55 | 2.26 | -0.26 | -0.01 | -0.07 | 0.19 | -0.07 | 0.03 | 0.06 | 0.00 | -0.05 |
|  | 8.0 | -0.02 | 0.56 | 2.33 | -0.26 | -0.01 | -0.07 | 0.20 | -0.07 | 0.03 | 0.06 | -0.01 | -0.05 |
| pY | 4.0 | 0.19 | -0.51 | 0.41 | 0.11 | 0.01 | 0.05 | -0.33 | -0.11 | -0.07 | -0.06 | -0.05 | -0.08 |
|  | 4.5 | 0.19 | -0.51 | 0.40 | 0.12 | 0.01 | 0.05 | -0.33 | -0.12 | -0.07 | -0.06 | -0.05 | -0.08 |
|  | 5.0 | 0.19 | -0.50 | 0.39 | 0.12 | 0.01 | 0.05 | -0.33 | -0.12 | -0.07 | -0.06 | -0.05 | -0.08 |
|  | 5.5 | 0.21 | -0.52 | 0.33 | 0.10 | 0.02 | 0.07 | -0.34 | -0.12 | -0.08 | -0.06 | -0.05 | -0.07 |
|  | 6.0 | 0.21 | -0.53 | 0.29 | 0.09 | 0.03 | 0.09 | -0.34 | -0.13 | -0.08 | -0.06 | -0.05 | -0.07 |
|  | 6.5 | 0.25 | -0.53 | 0.23 | 0.08 | 0.04 | 0.11 | -0.34 | -0.14 | -0.08 | -0.06 | -0.05 | -0.07 |
|  | 7.0 | 0.24 | -0.56 | 0.19 | 0.07 | 0.05 | 0.12 | -0.35 | -0.14 | -0.08 | -0.06 | -0.06 | -0.07 |
|  | 7.5 | 0.24 | -0.56 | 0.17 | 0.06 | 0.05 | 0.12 | -0.35 | -0.14 | -0.08 | -0.06 | -0.06 | -0.07 |
|  | 8.0 | 0.24 | -0.57 | 0.17 | 0.07 | 0.05 | 0.12 | -0.35 | -0.14 | -0.08 | -0.06 | -0.06 | -0.07 |

# theoretical values, extrapolated for the fully deprotonated state
